# Commencements

**Published:** 1891-04

**Authors:** 


### COMMENCEMENTS.

---

The commencement exercises of the two medical colleges in this city were held last month. The thirty-ninth annual commencement of the Atlanta Medical College took place at the opera house, March 2d. The report of the Proctor, Dr. W. S. Kendrick, showed that the session just closed had been the most successful one in the history of the College. There had been present during the term 163 students, of whom 80 were graduating. The degrees upon these were conferred by Hon. N. J. Hammond, President of the Board of Trustees. Following are the honor men: First, Dr. J. A. Weaver; second, Dr. B. S. Burton; third, Dr. L. B. Bouchelle, Jr. Each of these gentlemen was the recipient of a handsome gold medal.

Prof. Chas. A. Lane, of Georgia, delivered a bright and entertaining address to the graduating class.

Dr. Frank Park, of LaGrange, a young man of marked ability, was the valedictorian of the class. Dr. W. R. Slack, of LaGrange, then presented Dr. Clarence Johnson with a beautiful gold-headed cane, as a mark of the esteem in which he was held by the class of '91.

The twelfth annual commencement of the Southern Medical College was held at DeGive's Opera House, March 4th. The Dean of the Faculty, Dr. Wm. Perrin Nicolson, presided. In his report, Dr. Nicolson said that the purpose of the founders, to make this a school whose tendency would be to elevate the standard of medical teaching in the South, was being carried out, and that the work of the session just closing was encouraging. There had been in attendance in the two departments (medical and dental) 207 students, 102 of whom were in the medical department.

The graduating class, 36 in number, were then presented with their diplomas by Dr. Thos. S. Powell, the President of the College. Mr. Chas. A. Read, of Atlanta, delivered an able and witty address to the graduates.

The valedictorian of the class was Dr. J. McDiarmid, of Georgia.

The prizes were awarded as follows: First, Dr. T. H. Fritts, Tenn.; second, Dr. D. C. Rumph, Texas; third, Dr. J. W. Price, Virginia. The demonstrator's prize, a handsome case of instruments, was given to Dr. J. McDiarmid.

The Southern College has now in contemplation the erection of a new college building, which will greatly enlarge its facilities for doing good work.

